# Hexokinase is a key regulator of energy metabolism and ROS activity in insect lifespan extension

**DOI:** 10.18632/aging.100885

**Published:** 2016-02-07

**Authors:** Xian-Wu Lin, Wei-Hua Xu

**Affiliations:** ^1^ State Key Laboratory of Biocontrol, School of Life Sciences, Sun Yat-Sen University, Guangzhou 510006, China

**Keywords:** hexokinase, developmental arrest, transcription factor, gene regulation, Helicoverpa armigera

## Abstract

Developmental arrest (diapause) is a ‘non-aging’ state that is similar to the *Caenorhabditis elegans* dauer stage and *Drosophila* lifespan extension. Diapause results in low metabolic activity and a profound extension of insect lifespan. Here, we cloned the *Helicoverpa armigera* Hexokinase (HK) gene, a gene that is critical for the developmental arrest of this species. HK expression and activity levels were significantly increased in nondiapause-destined pupae compared with those of diapause-destined pupae. Downregulation of HK activity reduced cell viability and delayed pupal development by reducing metabolic activity and increasing ROS activity, which suggests that HK is a key regulator of insect development. We then identified the transcription factors Har-CREB, -c-Myc, and -POU as specifically binding the Har-HK promoter and regulating its activity. Intriguingly, Har-POU and -c-Myc are specific transcription factors for HK expression, whereas Har-CREB is nonspecific. Furthermore, Har-POU and -c-Myc could respond to ecdysone, which is an upstream hormone. Therefore, low ecdysone levels in diapause-destined individuals lead to low Har-POU and -c-Myc expression levels, ultimately repressing Har-HK expression and inducing entry into diapause or lifespan extension.

## INTRODUCTION

Environmental conditions are not always suitable for growth and development; thus, to adapt to adverse environmental conditions, most insect species have evolved a special stage of developmental arrest called diapause. Diapause may occur during various developmental stages e.g., embryonic, larval, pupal or adult; however insects typically enter diapause only once during their life cycle. For example, upon short day length signaling, *Helicoverpa armigera* (cotton bollworm) pupae will enter diapause for months, which results in the extreme extension of their lifespan compared with their nondiapause counterparts that experience long day lengths [1]. Diapause is a ‘non-aging’ state, similar to the *Caenorhabditis elegans* dauer stage [2] and *Drosophila* lifespan extension [3]; these stages exhibit the same low metabolic activity phenotype, which indicates that *H. armigera* is an excellent model for research aimed at understanding the mechanism underlying lifespan extension.

Previous studies have demonstrated that pupal diapause is caused by a halt in prothoracicotropic hormone (PTTH) release in the brain and the subsequent failure of the prothoracic glands to synthesize the steroidal hormone 20-hydroxyecdysone (20E) that is required for continuous development [4]. Recently, Xu et al. [5] found that low 20E levels suppress fat body production of tricarboxylic acid (TCA) intermediates, and low levels of TCA intermediates in the hemolymph lead to low brain TCA cycle activity, i.e., low brain activity leads to pupal diapause entry. This result indicated that TCA cycle activity is a key point of control over brain metabolic activity; however, how TCA activity is regulated in the diapause brain is unclear.

Hexokinase (HK) (ATP: D-hexose-6-phospho-transferase, EC 2.7.1.1) converts glucose to glucose-6-phosphate and is the first rate-limiting enzyme in glycolysis. In most organisms, glucose is the most important HK substrate, and the resulting glucose-6-phosphate is then converted to pyruvate for use in the TCA cycle. HK plays a central role in regulating energy metabolism *via* the TCA cycle [5]. Injection of a HK inhibitor, 2-Deoxy-glucose (DOG), delays *H. armigera* pupal development [4]; therefore, we speculate that HK may be a key factor in the regulation of energy metabolism in the *H. armigera* brain during pupal diapause.

To understand the molecular mechanism regulating pupal diapause in the brain, we cloned HK cDNA from *H. armigera* brains and investigated its expression in pupal brains of diapause- and non-diapause-destined individuals. HK expression and activity levels were significantly reduced in diapause-destined individuals, which indicates that HK is a critical factor in the reduction of metabolic activity for diapause initiation. Together, the downregulation of HK expression and activity could cause increased activity of reactive oxygen species (ROS), central regulators in lengthening lifespan, to induce diapause [6, 7]. Furthermore, we demonstrated that the POU, c-Myc, and cAMP responsive element-binding protein (CREB) transcription factors regulate HK transcription, and 20E can activate HK expression by activating these transcription factors. Therefore, HK plays a critical role in controlling energy metabolism and ROS activity in the brain for diapause or lifespan extension.

## RESULTS

### Har-HK cDNA isolation and molecular characterization

**Figure 1:**
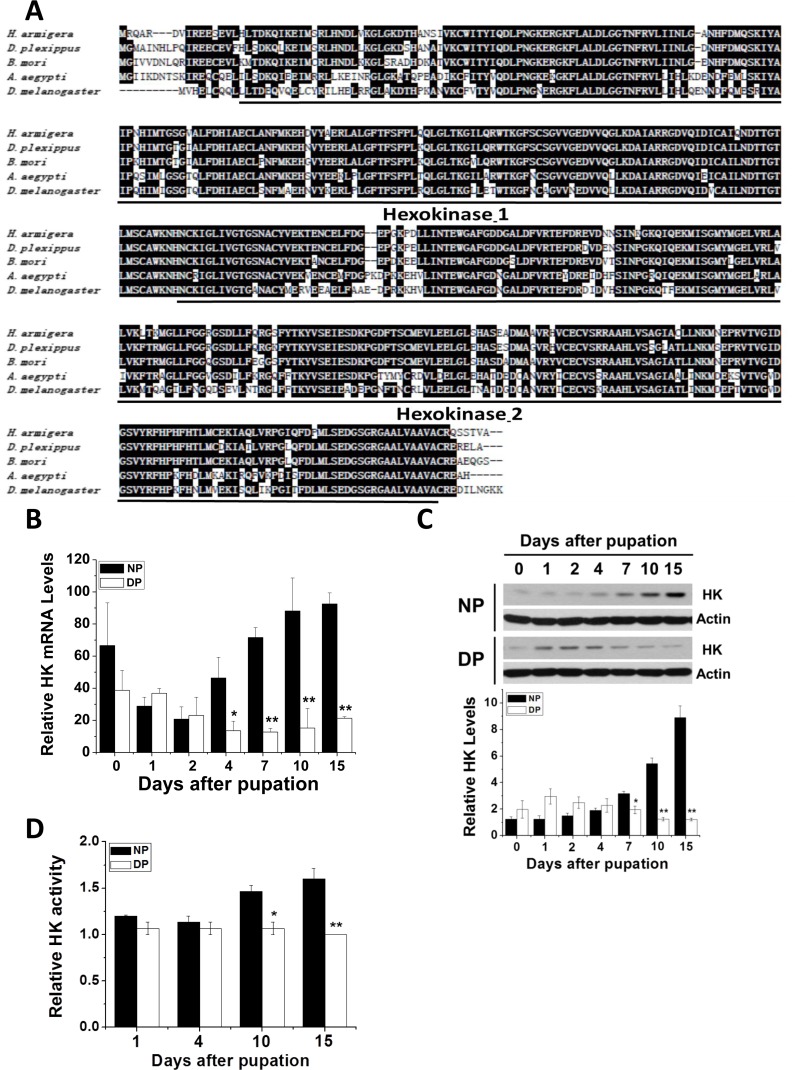
HK protein sequence alignment and Har-HK expression pattern (**A**) Amino acid homology comparison between Har-HK and other known HKs; Hexokinase-1 and -2 are two highly conserved domains; black shading denotes ≥ 60% sequence identity. *D. plexippus*, EH-J75730.1; *B. mori*, XP-004932651.1; *A. aegypti*, XP-001660031.1; *D. melanogaster*, NP_-_727350.1. (**B**) Har-HK mRNA expression through development. Total RNA was extracted from the brains of nondiapause- and diapause-destined pupae and used for qPCR. (**C**) Har-HK protein expression through development. Brain proteins (20 μg) were extracted for western blotting analysis. DP, diapause-type pupae; NP, nondiapause-type pupae. Expression levels were quantified using ImageJ software and normalized to Har-actin levels (5 μg). (**D**) Brain Har-HK activity. Har-HK activity levels in the brains of nondiapause- and diapause-destined pupae were assayed. Each point **(B-D)** represents the mean ± S.D. of three independent replicates. The * denotes *p* < 0.05, and ** denotes *p* < 0.01 as determined by the independent *t*-test.

Using the known *B. mori*, *D. melanogaster*, and *Danaus plexippus* HK cDNA sequences, we synthesized degenerate primers to amplify HK cDNA from *H. armigera* (Har-HK cDNA). A 530 bp cDNA fragment was obtained by RT-PCR, and full-length cDNA was amplified using RACE. Full-length HK cDNA is 1851 bp (GenBank accession no. KR780750.1), contains the 5′- and 3′-untranslated regions (188 and 317 bp, respectively), and an open reading frame (ORF) encoding a 450 amino acid protein. The amino acid sequence contains two highly conserved domains and shares high identity with other HKs: *D. plexippus* (92%), *B. mori* (90%), and *D. melanogaster* (72%) (Fig. 1A); therefore, we refer to this cDNA as Har-HK cDNA.

### Har-HK expression and activity associated with pupal development

Nondiapause- and diapause-destined pupae were incubated at 20°C. Nondiapause pupae develop into adults within 23–24 days, whereas diapause-destined pupae enter diapause within approximately 8–10 days and their pupal lifespan is greater than 3 months. Consequently, we focused on diapause initiation from day 0 to day 10. To test whether Har-HK participates in diapause initiation, we performed qPCR and western blotting analysis to examine Har-HK gene expression in the pupal brains of diapause- and nondiapause-destined individuals (Fig. 1B). Har-HK mRNA expression levels were similar among the groups between 0 and 2 days, but were significantly greater in the brains of nondiapause-destined pupae than in diapause-destined pupae between 4 and 15 days.

Western blotting analysis using the Har-HK polyclonal antibody revealed that Har-HK protein expression was similar to its mRNA expression (Fig. 1C). Similar protein expression levels were observed between 0 and 4 days in both groups; however, protein expression was significantly greater in nondiapause-destined pupal brains between 7 and 15 days.

As HK functions in glycolysis, we investigated its activity in pupal brains (Fig. 1D). The changes in activity were similar to those of mRNA and protein expression levels: HK activity was greater in nondiapause individuals compared with diapause individuals at 10 and 15 days after pupation, indicating that diapause individuals require low HK activity. These results demonstrate that Har-HK expression and activity levels are reduced in diapause-destined pupae, suggesting that Har-HK may be a key factor in insect diapause induction.

### Suppression of Har-HK delayed development by decreasing metabolic activity and increasing ROS activity

**Figure 2:**
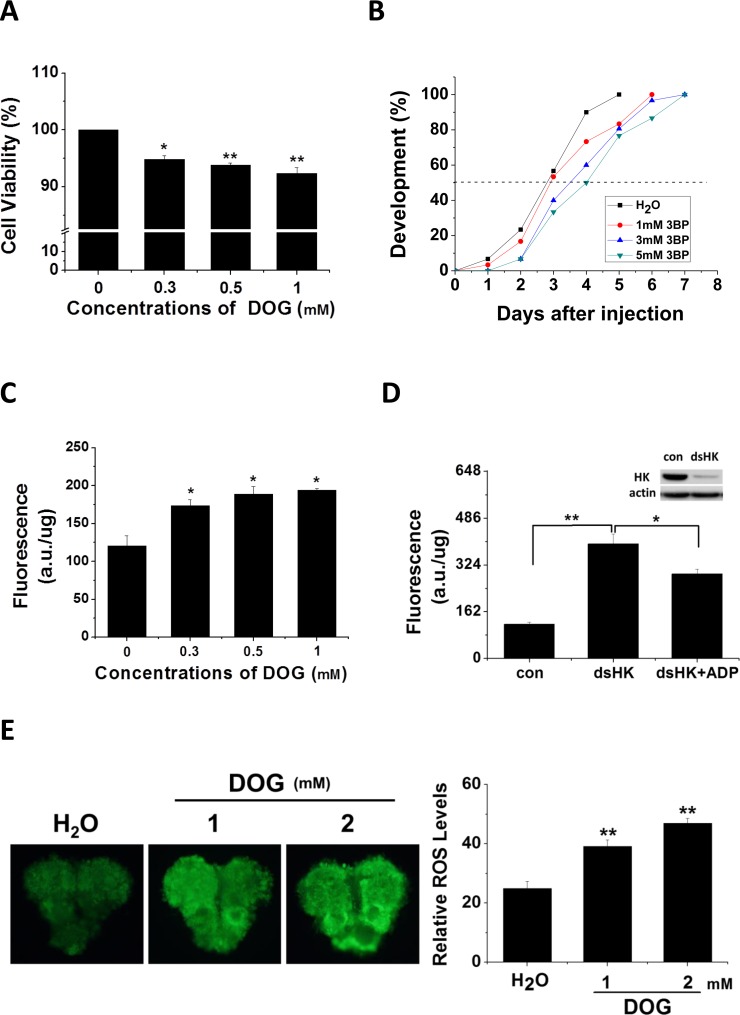
Inhibitor effects on cell viability, developmental delay, and ROS activity (**A**) Cell viability changes. HzAm1 cells were treated with various concentrations of DOG for 48 h, and viable cells were measured using the MTT assay; the values represent the mean ± S.D. of at three independent experiments. (**B**) Developmental delay caused by 3BP injection. Day 1 nondiapause-destined pupae were injected with 3 μl of 3BP at various concentrations (1 mM 3BP, *n* = 30; 3 mM 3BP, *n* = 30; and 5 mM 3BP, *n* = 30), and pupal stemmata were examined as a marker for pupal development. As a control, pupae were also injected with 3 μl of H_2_O (*n* = 30). (**C**) Effects of DOG on ROS activity. HzAm1 cells were treated with DOG for 24 h and fluorescence was measured. (**D**) ROS activity changes due to Har-HK knockdown. HzAm1 cells were transfected with dsGFP (con) or dsHK for 36 h and then treated with 1 mM ADP for 12 h; each point represents the mean ± S.D. of three independent replicates. (**E**) Effects of DOG on brain ROS activity. Day 1 nondiapause-destined pupae were injected with 3 μl of DOG (*n* = 5), and fluorescence was then measured. The * denotes *p* < 0.05, and ** denotes *p* < 0.01 as determined by the independent *t*-test.

To assay HK function in insect development, two HK inhibitors, DOG and 3-Bromopyruvic acid (3BP), were either added to cell culture medium or injected into pupae, respectively. Cells were treated with various concentrations of DOG and assayed for viability using the 3-(4, 5-dimethyl-2-thiazolyl)-2, 5-diphenyl-2-H-tetrazolium bromide (MTT) method (Fig. 2A). After 48 h of treatment with DOG, cell activity was significantly reduced, and viability decreased with increasing concentrations of DOG. We then injected day 1 nondiapause pupae with 3BP and monitored pupal stemmata migration, finding that pupal development was delayed 1–1.5 days (Fig. 2B). These results indicate that Har-HK inhibition results in low metabolic activity and developmental delay, which suggests that Har-HK controls energy metabolism to regulate insect development.

Previous studies have demonstrated that ROS play crucial roles in cell cycle arrest and lifespan extension in *C. elegans* [8, 9], and HK knockdown leads to increased ROS activity [10, 11]. To clarify whether reduced Har-HK activity in diapause-destined pupal brain affects ROS levels, we treated cells with various concentrations of DOG and observed that ROS levels were significantly increased in treated cells compared with the control cells (Fig. 2C). Using dsRNA-mediated knockdown of HK in the culture cells, we observed a significant increase in the ROS levels and partial rescue upon the addition of ADP (Fig. 2D). Similarly, DOG was injected into day-1 nondiapause pupae, which resulted in significantly increased ROS levels (Fig. 2E). These results suggest that reduced Har-HK activity can induce increases in ROS levels *in vivo* and *in vitro*.

### Characterization of Har-HK promoter transcription factor-binding sites

**Figure 3:**
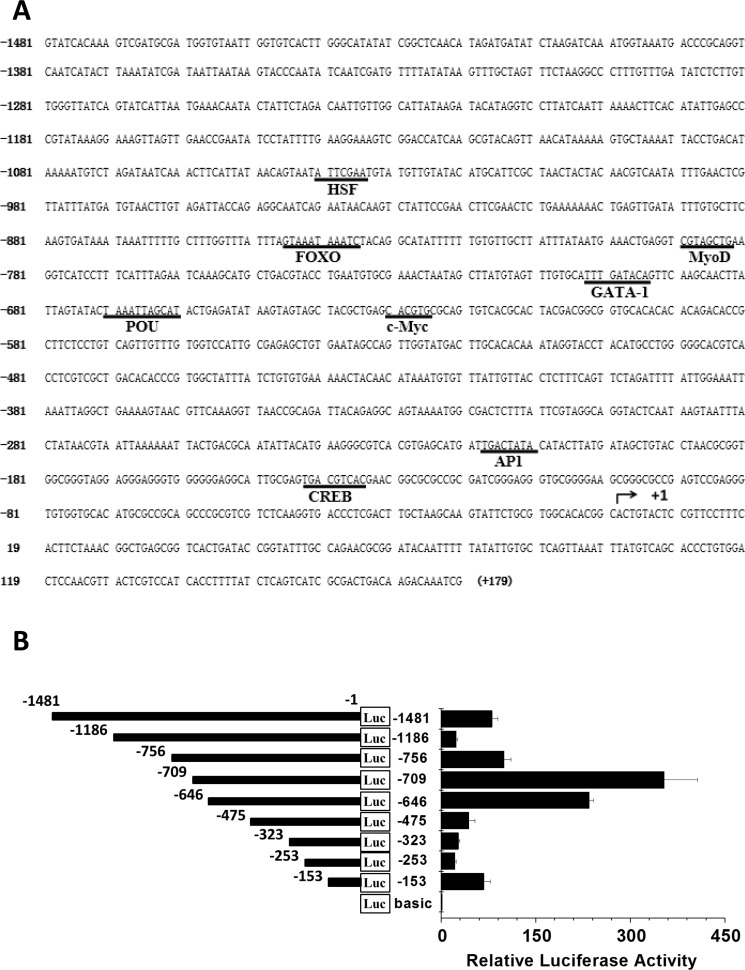
Har-HK promoter region sequence and activity (**A**) Har-HK promoter region nucleotide sequence; nucleotide positions are numbered relative to the predicted transcription start site (+1). The potential consensus sequences for regulatory elements and transcription factor-binding sites are underlined. (**B**) HK promoter deletion analysis. Diagram of deletion constructs covering various Har-HK promoter regions that were fused to a luciferase reporter gene and then transfected into HzAm1 cells. The pGL3-basic plasmid was used as a negative control, and each point represents the mean ± S.D. of three independent replicates.

Har-HK expression in diapause-destined pupal brains is regulated at the level of transcription as described above. To characterize the regulation of Har-HK gene expression, a 1481 bp fragment of the Har-HK gene 5′-upstream region was cloned using the genome walking technique [12] and then sequenced. The promoter was examined for potential regulatory element consensus sequences using the TFSEARCH website (www.cbrc.jp/research/db/TFSEARCH.html) [13]; several potential transcription factor-binding sites, including POU, c-Myc, CREB, AP4, MyoD, FoxO, GATA-1 and HSF, are shown in Figure 3A.

We then cloned nine Har-HK promoter truncations into a pGL3-basic luciferase reporter vector and assayed promoter activity to determine whether the potential cis-elements regulated Har-HK transcription (Fig. 3B). These constructs were co-transfected into HzAM1 cells with the pRL-TK plasmid as an internal control for transfection efficiency. Promoter activities were measured using a dual-luciferase reporter system; strong luciferase activity signals were detected when the HzAM1 cells were transfected with the −1 to −153 bp, −1 to −646 bp, and −1 to −709 bp regions.

**Figure 4:**
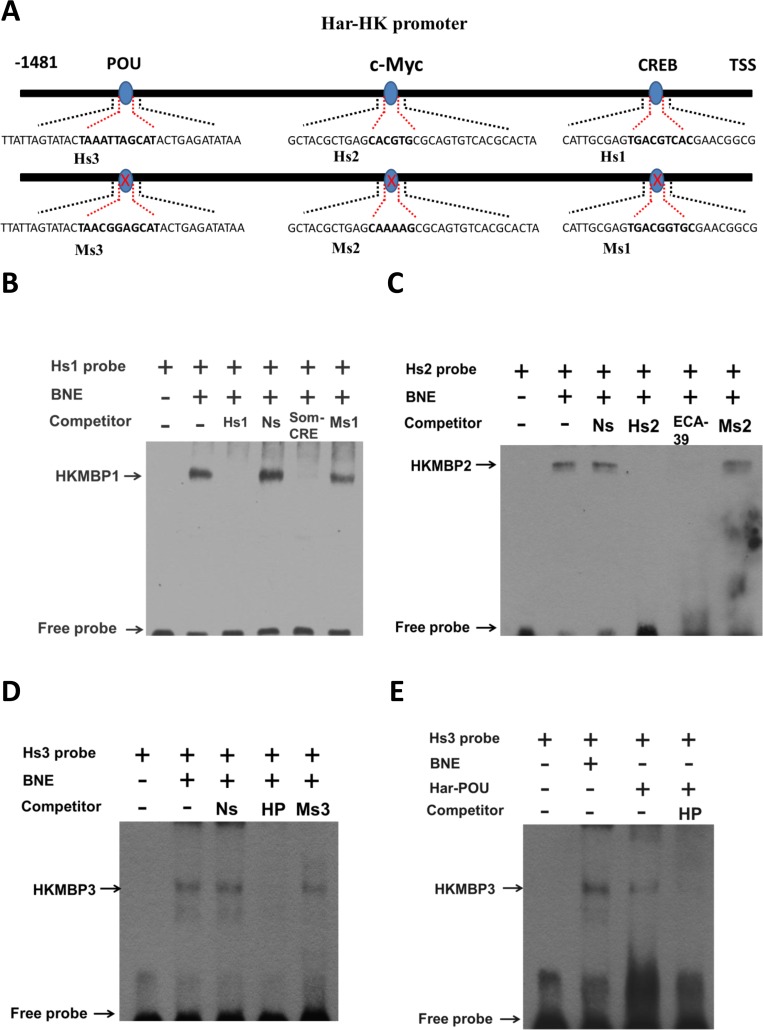
Electrophoresis mobility shift assays (EMSAs) using the activating region (**A**) Schematic drawing of EMSA probes. The Hs1, Hs2, and Hs3 probes contain the Har-HK promoter CREB-, c-Myc- and POU-binding regions (bolded), respectively. Three probes were mutated within the CREB- (Ms1), c-Myc- (Ms2) or POU-binding sites (Ms3); mutant regions are bolded. TSS: transcriptional start site; (**B**) A CRE-binding protein binds Hs1. The Hs1 probe was incubated with 5 μg of brain nuclear extract. BNE, brain nuclear extract; Ns, nonspecific competitor; Smo-CRE, rat somatostatin gene CRE-binding sequence; Ms1, CREB-binding site mutation; (**C**) c-Myc protein binds Hs2. The Hs2 probe was incubated with 5 μg of brain nuclear extract. ECA39, teratocarcinoma cell ECA39 gene c-Myc-binding sequence; Ms2, c-Myc-binding site mutation. (**D**) POU protein binds Hs3. The Hs3 probe was incubated with 5 μg of brain nuclear extract. HP: Har-DH-PBAN gene Har-POU-binding site; Ms3: POU-binding site mutation; (**E**) The *in vitro*-expressed Har-POU bind Hs3. The Hs3 probe was incubated with 5 μg of brain nuclear extract or 0.1 μl *in vitro*-expressed Har-POU. HKMBP1, 2, and 3: HK-modulating binding proteins1, 2, and 3, respectively.

A binding site for the CREB transcription factor is present in the fragment −1 to −153 bp. High activity in the −1 to −646 bp fragment is located between −475 and −646, which encompasses a c-Myc-binding site. The highest activity for the −1 to −709 bp fragment was observed between −646 and −709, which covers the POU- and GATA-1-binding sites, suggesting that these transcription factors may regulate Har-HK expression. To identify the cis-elements conferring promoter activity, three EMSA probes (Hs1, Hs2, and Hs3) and three mutated probes (Ms1, Ms2, and Ms3) were synthesized (Fig. 4A). A biotin end-labeled Hs1 probe containing the predicted CRE-binding site produced a distinct shift with pupal brain nuclear protein extract; we named this factor Har-HK modulating binding protein 1 (HKMBP1) (Fig. 4B). Incubation of the Hs1 probe with a 100-fold excess of unlabeled Hs1 completely eliminated the shift, whereas the shift could not be eliminated by an unrelated probe (nonspecific probe, Ns). The HKMBP1 shift also remained when an unlabeled probe containing a CRE-binding site mutation (Ms1) was used as a competitor. To further verify that HKMBP1 was a CRE-binding protein, the rat somatostatin CRE-binding site (Som-CRE) was used as a competitor [14], and completely eliminated the shift. Together, these results indicate that HKMBP1 is a CRE-binding protein and specifically binds to the CRE-binding region of the Har-HK promoter.

Using a biotinylated oligonucleotide probe containing a c-Myc-binding site (Hs2), a specific shift (HKMBP2) was detected upon incubation with nuclear protein extract (Fig. 4C), and this shift could not be eliminated using either the Ns probe or a probe containing a c-Myc-binding site mutation (Ms2). To further verify that HKMBP2 is c-Myc protein, the c-Myc-binding sequence of the teratocarcinoma cell ECA39 gene [15] and unlabeled probe (Hs2) were used as competitors and completely eliminated the shift. These results indicate that HKMBP2 is likely to be the c-Myc that specifically binds the c-Myc-binding region of the Har-HK promoter.

The Hs3 probe containing a POU-binding site exhibited a specific shift, HKMBP3 (Fig. 4D), which could not be eliminated by the Ns probe or the probe containing a POU-binding site mutation (Ms3). To confirm that HKMBP3 is the POU protein, the Har-DH-PBAN (HP) gene POU-binding site was used as a competitor [16] and was able to completely eliminate the shift. *In vitro*-expressed Har-POU could interact with Hs3 probe and exhibited identical mobility as HKMBP3 when incubated with nuclear extracts (Fig. 4E). Together, these results indicate that HKMBP3 is the transcription factor POU and that Har-POU can specifically bind the HK promoter.

### Har-CREB and -POU, developmental expression and ChIP assays

**Figure 5:**
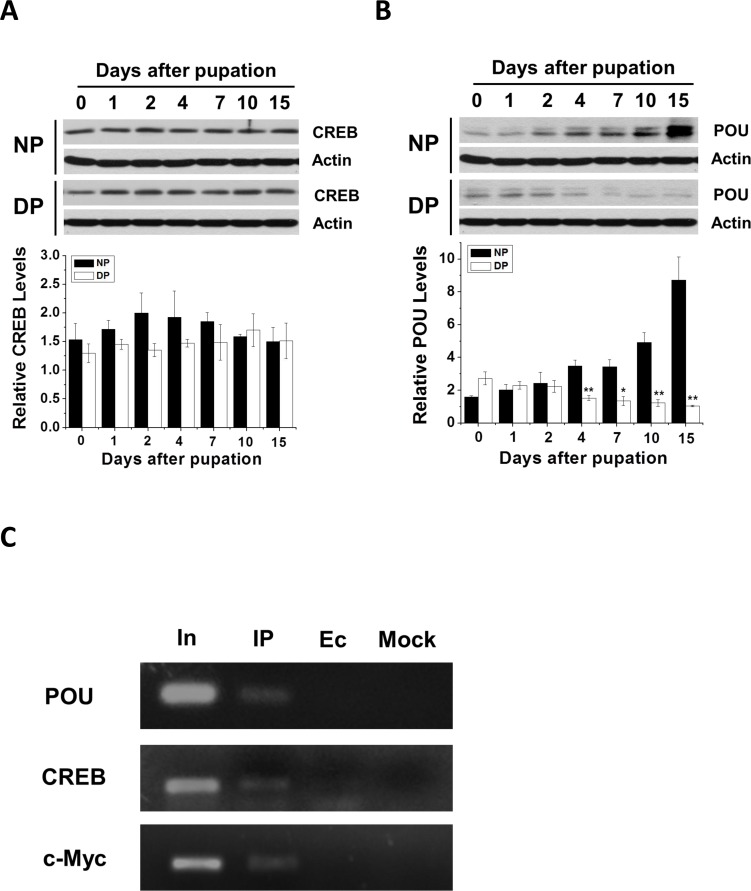
Developmental expression and ChIP assay (**A**) and (**B**) Har-CREB and -POU expression patterns during pupal stages. Brain proteins (20 μg) were extracted and used for Western blotting analysis. DP, diapause-type pupae; NP, nondiapause-type pupae. (**C**) Har-POU, -CREB and -c-Myc ChIP assays testing Har-HK promoter binding. In, input; IP, DNA immunoprecipitated using anti-Har-POU, -CREB and -c-Myc antibodies; Ec, empty control immunoprecipitated using pre-immune serum; Mock, mock control immunoprecipitated using anti-Har-actin antibody.

To further verify that these transcription factors regulate Har-HK expression, we cloned Har-CREB using the strategy previously described for Har-HK (see Supplementary Figure 1), Har-POU and -c-Myc [16, 17]. Polyclonal antibodies against Har-CREB and -POU were generated to investigate Har-CREB and -POU expression in nondiapause- and diapause-destined pupal brains; Har-c-Myc expression in *H. armigera* pupal brains was previously reported by Chen and Xu [17]. Western blotting analysis indicated that Har-CREB protein levels were not significantly different (Fig. 5A); however, Har-POU protein levels in nondiapause pupal brains were initially similar to those of diapause individuals (0–2 days), but were significantly increased later (4–15 days; Fig. 5B). This pattern was similar to that of Har-c-Myc expression [17]. These results showed that the expression patterns of Har-POU and -c-Myc are consistent with that of Har-HK, which suggests that Har-HK can respond to changes in Har-POU and -c-Myc and that Har-POU and -c-Myc function as transcription factors that specifically regulate Har-HK expression for diapause or development. Furthermore, Har-CREB may also regulate Har-HK expression as a basic transcription factor, but it is not specific to either type of individual.

To test transcription factor binding to the Har-HK promoter *in vivo*, we measured Har-CREB, -c-Myc, and -POU binding to the Har-HK promoter in day 10 pupal brains using ChIP assays. PCR products were detected when anti-Har-POU, -CREB, and -c-Myc antibodies were used, but no obvious bands were present in the negative control (Fig. 5C). These results suggest that three transcription factors can bind to the Har-HK promoter *in vivo*.

### Changes in Har-POU, -CREB, and -c-Myc expression modulate Har-HK promoter activity

**Figure 6:**
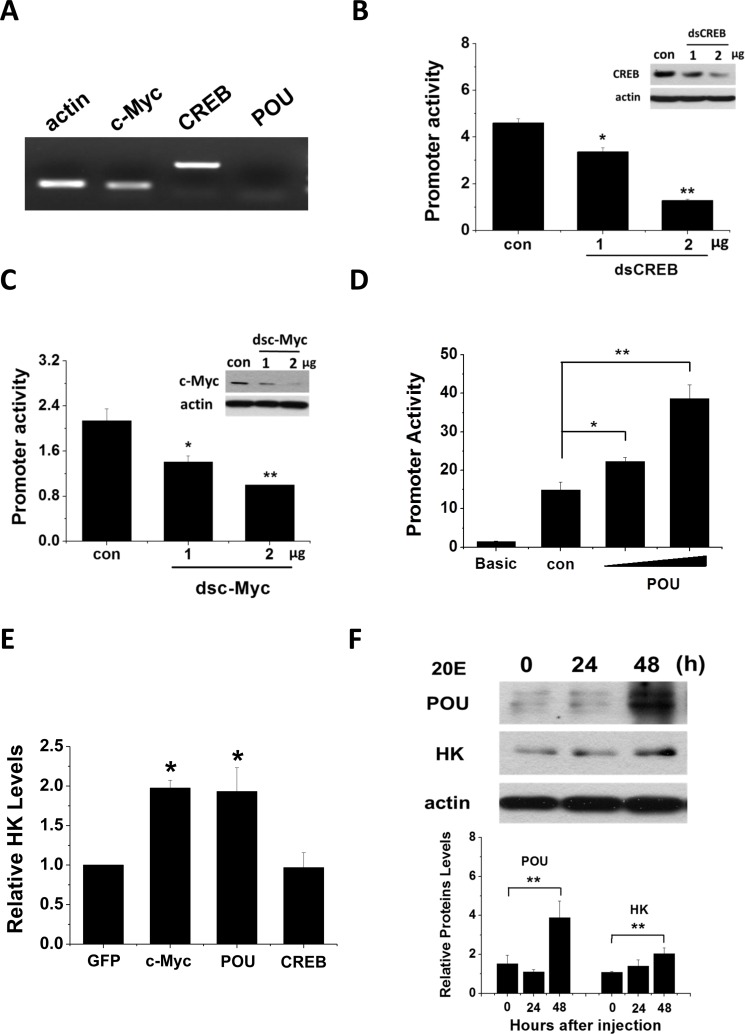
Har-CREB, -c-Myc and -POU regulate Har-HK promotor activity and gene expression (**A**) Har-CREB, -c-Myc and -POU expression in HzAm1 cells. Total RNA was extracted from cells and reverse transcribed to generate cDNA for the amplification of c-Myc, CREB, POU, and an acting control. (**B**) and (**C**) RNA interference directed against Har-CREB and –c-Myc reduces Har-HK promoter activity. The Har-HK promoter reporter plasmid (0.2 μg) was co-transfected with various amounts of Har-CREB or -c-Myc dsRNA (0.2 and 0.4 μg); 0.4 μg of dsGFP was used as a control. Luciferase activity was detected 48 h following transfection. (**D**) Har-POU activated the Har-HK promotor. HzAm1 cells were co-transfected with Har-HK promoter reporter plasmid (0.2 μg) and luciferase reporter vector (pGL3)-basic with or without 0.2 or 0.4 μg of Har-POU plasmid. Luciferase activity was detected 48 h following transfection. (**E**) Changes in Har-HK mRNA due to Har-CREB, -c-Myc and -POU overexpression. HzAm1 cells were transfected with plasmids encoding recombinant Har-CREB, -c-Myc, -POU, and GFP, and Har-HK mRNA was detected by qPCR using *actin* as an internal standard. **(F)** Ecdysteroid (20E) regulates Har-HK expression by controlling Har-POU protein levels. 1 μg of 20E was injected into day 20 diapausing pupae, and changes in brain Har-HK and -POU protein levels were detected at 24 h and 48 h following injection. Brain proteins (20 μg) were separated and detected with the appropriate antibodies. Con, injection of 2 μl ethanol; 20E, injection of 2 μl 20E (0.5 μg/μl, dissolved in ethanol). The western blotting bands were quantified using ImageJ software and normalized to Har-actin levels. Each point represents the mean ± S.D. of three independent replicates. The * denotes a *p* value < 0.05, and ** denotes a *p* value < 0.01 as determined by the independent *t*-test.

We used RT-PCR to examine Har-POU, -CREB, and -c-Myc gene expression in the HzAm1 insect cell line. Har-CREB and -c-Myc were highly expressed; in contrast, Har-POU expression was undetectable (Fig. 6A). Consequently, we assayed Har-HK promoter regulation by Har-POU overexpression, and dsCREB and dscMyc knockdown in HzAm1 cells. When the Har-HK promoter was co-transfected with dsCREB or dscMyc, it was clear that Har-CREB and -c-Myc expression levels were reduced by RNAi in HzAm1 cells, leading to reduced Har-HK promoter activity (Figs. 6B and 6C). HzAm1 cells were co-transfected with Har-POU fused to the pIZ/V5-GFP plasmid and the Har-HK promoter. In these cells, luciferase activity was absent compared to the background, and low activity was observed only in cells that were transfected with the Har-HK promoter (Fig. 6D). In contrast, when the Har-HK promoter and Har-POU were co-transfected into HzAm1 cells, the forced expression of Har-POU significantly activated the Har-HK promoter.

Finally, we transfected HzAm1 cells with Har-CREB, -POU, and -c-Myc, and investigated HK mRNA expression by qPCR. From this analysis, we found that transfection with Har-POU and -c-Myc, but not Har-CREB, resulted in significantly increased HK mRNA expression (Fig. 6E).

Together, these results indicate that Har-POU overexpression can activate the Har-HK promoter, while reduced Har-CREB and -c-Myc expression reduces Har-HK promoter activity. These results suggest that Har-POU and -c-Myc play key roles in regulating insect development by activating HK expression.

### POU activates Har-HK expression through 20E

Ecdysone is well known as one of the most important hormones in insect development. Moreover, POU and c-Myc expression patterns resemble the change in ecdysone titers: their levels gradually increase during pupal development in nondiapause pupae but remain at low levels in diapause-destined pupae. Recent work demonstrated that Har-c-Myc expression is ecdysone-responsive [17]; consequently, we speculated that ecdysone may also regulate Har-POU for Har-HK expression and activity. To test this idea, we injected day 20 diapausing pupae with 1 μg of 20E to promote development and found that Har-POU protein gradually increased from 24 h to 48 h after injection (Fig. 6F); simultaneously, Har-HK protein expression also gently increased. This *in vivo* experiment clearly demonstrates that Har-POU is responsive to ecdysone exposure, suggesting that ecdysone may be an upstream signal that regulates Har-POU and -c-Myc expression to control Har-HK.

## DISCUSSION

Diapause is an environmentally preprogrammed period of developmental arrest and is characterized by metabolic depression [18]. Previous work demonstrated that diapause-destined pupae possess low TCA cycle activity, which results in low brain activity and induces diapause entry [5]. This result indicated that sugar metabolism plays a crucial role in diapause or lifespan extension individuals; however, the regulatory mechanisms remained unknown.

In this work, we focused on HK, the first enzyme in glycolysis, with respect to the regulation of pupal development or diapause. Significant differences in Har-HK mRNA and protein levels, as well as activity levels were observed between diapause- and nondiapause-destined pupal brains. Cell activity and pupal development were blocked by the HK inhibitors DOG and 3BP, indicating that low Har-HK activity leads to low metabolic activity in diapause-destined individuals. Moreover, our results demonstrate that the downregulation of Har-HK expression and activity results in an increased accumulation of ROS. Recent studies have demonstrated that impaired insulin/IGF signaling or inhibition of mitochondrial respiration can extend the lifespan *via* the increased accumulation of ROS [7, 19, 20]. Thus, ROS is likely a key regulator of lifespan extension. Our results suggest that Har-HK plays two key roles in insect diapause: i) Har-HK acts as a molecular switch to directly control energy production for metabolism, and ii) Har-HK down-regulation causes increased ROS levels and induces individuals to enter diapause or lifespan extension.

Previous studies have demonstrated that the CREB, STAT3, and JunD transcription factors regulate mammalian HK expression [21–25]; however HK regulation remained unclear in insects. In this report, we identified three transcription factors, Har-CREB, -POU, and -c-Myc, that regulate Har-HK expression. These roles are supported by (i) competition for binding to Har-CREB, -POU, and -c-Myc using specific probes containing known CREB, POU, and c-Myc-binding sites [13–15]; (ii) Har-CREB, -POU, and -c-Myc binding to the Har-HK promoter *in vivo*; and (iii) changes in Har-POU, -CREB, and -c-Myc levels modulated Har-HK promoter activity. These three transcription factors play different roles in the regulation of Har-HK expression. Har-CREB has a low activity towards the Har-HK promoter (Fig. 3B), as its overexpression does not affect HK mRNA levels, and its expression is similar in diapause and nondiapause pupal brains. Consequently, we speculate that Har-CREB may be a basic transcription factor for HK expression, but its function is unrelated to insect diapause. In contrast, Har-POU and -c-Myc are highly expressed in nondiapause individuals, are expressed at low levels in diapause individuals, and can effectively activate the Har-HK promoter. These results indicate that Har-c-Myc and -POU are specifically expressed in nondiapause individuals during insect development and activate the Har-HK promoter. We suggest that Har-POU and -c-Myc are specific transcription factors for high Har-HK expression levels in nondiapause individuals. Additionally, the region containing the highest promoter activity (−646 to −709) contains two putative *cis* elements that bind POU and GATA-1. We were unable to isolate *H. armigera* GATA-1 cDNA using RACE, as GATA-1 cDNAs have only been reported in *D. melanogaster*, *Aedes aegypti*, and *Tribolium castaneu*; whether GATA-1 regulates Har-HK expression remains to be elucidated in the future.

It is well established that ecdysone promotes pupal-adult development but that low ecdysone levels induce pupal diapause. Here, we demonstrated that as previously reported for Har–cMyc, Har-POU can respond to ecdysone [17]. Ecdysone is an upstream signal that regulates Har-POU and -c-Myc expression for Har-HK activity; therefore, the Har-HK signaling pathway inducing insect development acts through ecdysone, Har-POU, -c-Myc and -HK.

**Figure 7:**
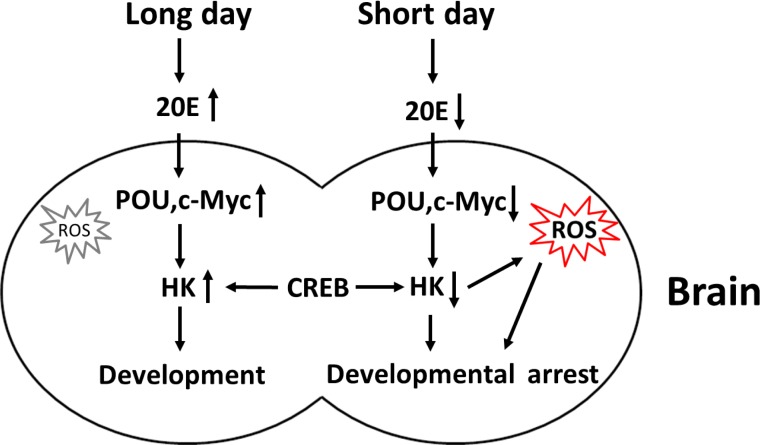
Schematic representation of Har-HK regulation during insect diapause and development Long day lengths trigger increased ecdysone (20E) levels, stimulating Har-POU and -c-Myc expression, and leading to high HK expression for development. In contrast, short day lengths repress ecdysone levels, resulting in low Har-POU and -c-Myc expression levels that lead to low Har-HK expression levels. Low HK expression levels cause low metabolic activity and increased ROS accumulation in diapause individuals. As a key diapause regulator, ROS induces entry into developmental arrest or lifespan extension.

The proposed model for the regulation of Har-HK expression is shown in Fig. 7. When short day lengths induce low ecdysone levels, Har-POU and Har-c-Myc transcription factor expression levels are reduced, leading to the repression of Har-HK promoter activity, and the subsequent low levels of Har-HK result in low metabolic activity in diapause individuals. Simultaneously, reduced Har-HK expression induces increased ROS activity, which is a diapause-determining factor, and the ROS induce insects to enter diapause. In contrast, long day lengths induce high ecdysone levels, activating Har-POU and -c-Myc expression and subsequently promoting Har-HK expression. High Har-HK expression levels cause high metabolic activity, promoting development.

## MATERIALS AND METHODS

### Animals

*H. armigera* larvae were reared on an artificial diet at 20°C under a photoperiod of L14:D10 (light : dark) to generate nondiapause pupae, and a L10:D14 photoperiod to generate diapause pupae. The development stages were synchronized at each molt by collecting new larvae or pupae. Pupal brains were dissected in ice-cold insect saline containing 0.75% NaCl and stored at −80°C until use.

### RNA extraction, DNA amplification, and rapid amplification of cDNA ends (RACE)

Total RNA was extracted from pupal brains as reported in Chen et al. [17], 1 μg of total RNA was reverse transcribed at 37°C for 1 h using M-MLV reverse transcription system (Promega, USA). One μl of reverse transcription product was added to 20 μl of the PCR reaction system, and amplification was performed with degenerate primers designed according to the conserved HK cDNA sequences from *Bombyx mori* (GenBank accession no. XP-004932651.1), *Danaus plexippus* (GenBank accession no. EHJ75730.1) and *Drosophila melanogaster* (GenBank accession no. NP_-_727350.1). PCR was performed under the following conditions: 5 min at 94°C; 30 cycles of 30 s at 94°C, 30 s at 42°C, and 60 s at 72°C; and 5 min at 72°C. A 530 bp of PCR product for HK was obtained and sequenced.

For 5′- and 3′-RACE, the first strand cDNA (Fs-cDNA) was synthesized using the SMART RACE cDNA amplification kit, according to the manufacturer's protocols (Clontech, USA). Two primers ACACGCACTCGCATACGTGGC and CACTCGCGT GCGACAATCC for HK 5′-RACE, and two primers CTCATCGTTGGCACTGGCAGC and GAGTTCGAC CGCGAAGTCGAC for HK 3′-RACE, were respectively synthesized. Using nest PCR, 5′- and 3′-HK cDNAs were respectively amplified as described in Xu [26].

### Developmental expression of Har-HK mRNA in the brain

The developmental expression of Har-HK mRNA was investigated using real-time quantitative PCR. First-strand cDNA was synthesized according to the procedure described above, and PCR was performed with primers (see Supplementary Table 1) in a Light Cycler480 (Roche, Switzerland) using SYBR Premix Ex Taq II (TaKaRa, China). *H. armigera rpL32* was used as an internal standard.

### Polyclonal antibody generation and western blot analysis

Recombinant Har-HK protein was expressed in *BL21* (DE3), purified on a Ni-NTA column and then used to generate polyclonal antibodies in rabbits.

Pupal brain proteins were extracted by homogenization in radioimmune precipitation assay buffer (50 mM Tris-HCl (pH 8.0), 150 mM NaCl, 1% Nonidet P-40, 0.1% SDS, 0.5% sodium deoxycholate, 1 mM PMSF, 1 mM EGTA, 5 mM NaF, and 10 mM Na_3_VO_4_). The lysates were then shaken in a rotary for 1 h at 4°C, and centrifuged at 12,000 *g* for 20 min at 4°C. The proteins were quantified using the Bradford method, and 5–20 μg samples were separated using 12% SDS-PAGE gels and transferred onto PVDF membranes (Millipore, USA). Non-specific binding was prevented using a solution of 5% milk without fat. HK, actin, CREB, c-Myc and POU antibody immunoreactivities were tested at 1:5000–1:10,000 dilutions and specific bands were detected with the SuperSignal West Pico chemiluminescent substrate (Thermo, USA).

### Har-HK activity assays

HK activity was measured as previously described using the total capacity for glucose phosphorylation of whole cell lysates [27]. Briefly, glucose-6-phosphate dehydrogenase converts HK-produced glucose-6-phosphate to 6-phosphogluconate and reduces NADP to form NADPH, which is spectrophotometrically monitored at 340 nm. Assays were performed in buffer containing 50 mM triethanolamine, 3.3 mM MgCl_2_, 20 mM glucose, 5.25 mM ATP, 0.75 mM NADP and 0.1 unit of glucose-6-phosphate dehydrogenase.

### Genome walking

The genomic DNA of *H. armigera* was extracted from the pupal brains according to the previous method [12, 28], and the DNA sample was then treated with Genome Walker^TM^ Universal Kit (Clontech, USA) according to the manufacturer's protocol. The primers for primary PCR HKW1 (5′-GGAGTCCACAGGGTGCTGAC-3′) and secondary PCR HKW2 (5′-GTATCAGTGACCGCTCAGCCG-3′) were designed based on Har-HK cDNA sequence. The samples were denatured at 94°C for 10 min, followed by a 30 cycles reaction 1) primary PCR: 94°C for 30 s; 58°C for 30 s; 72°C for 4 min; 2) secondary PCR: 94°C for 30 s; 60°C for 30 s; 72°C for 4 min. The PCR products were separated by 1.0% agarose gel electrophoresis, and then purified, ligated into the pMD18-T vector (TaKaRa, China) for sequencing.

### Cell culture, transfection and luciferase activity

HzAm1 cells from *Helicoverpa zea*, a close relative of *H. armigera*, were cultured at 27°C in Grace's insect cell culture media supplemented with 10% fetal bovine serum.

Transfections were performed using the FuGENE^®^ HD Transfection Reagent (Promega, USA) according to the manufacturer's instructions. Cells were suspended and plated in 96- or 24-well plates and cultured for 12 h. Plasmid DNAs were mixed with transfection reagent in a 1:3 ratio in sterile water (10 or 50 μl final volume), incubated at room temperature for 15 min, added to the wells, and the plates were gently shaken and then returned to incubator for 48 h.

The Dual-Luciferase^®^ Reporter Assay System (Promega, USA) was used to measure HK promoter activity as previously described [29]. Briefly, primers (see Supplementary Table 1) were used to amplify HK gene promoters of various lengths; the resulting fragments were digested with NheI and XhoI, and subcloned into similarly digested pGL3-basic vector. The resulting pGL3-HK vector was transfected with or without dsRNA targeting POU, CREB or c-Myc. The pRL-TK vector (Promega, USA) was used an internal control for variations in transfection efficiency. Luciferase activities were determined in triplicate in three separate experiments using a MikroWin2000 microplate luminometer (Mikrotex).

### Electrophoresis mobility shift assays (EMSAs)

Nuclear protein extracts were prepared from pupal brains using the NE-PER Nuclear and Cytoplasmic Extraction Reagents kit (Thermo, USA) according to the manufacturer's instructions. EMSAs were performed using the LightShift^®^ Chemiluminescent EMSA Kit (Pierce, USA). Briefly, 5 μg of nuclear proteins were incubated at room temperature for 20 min with 20 μl containing 50 ng/μl poly(dI-dC), 2.5% glycerol, 5 mM MgCl_2_, 0.05% NP40, and 20 fmol of biotin end-labeled probes. The reaction mixtures were then separated and transferred onto positively charged nylon membranes. The transferred DNA was then crosslinked to the membrane by exposure to UV light for 1 min (254 nm, 1200 mJ). The membrane was then incubated with a streptavidin-horseradish peroxidase conjugate and shifts were detected by enhanced chemiluminescence. For competition experiments, a 100-fold excess of unlabeled probe was incubated with the nuclear protein extract at room temperature for 20 min and then used for the above procedures.

### *In vitro* RNA knockdown

dsRNAs targeting HK, c-Myc, CREB and GFP were synthesized using the T7 RiboMAX express RNAi system (Promega, USA). Primers (see Supplementary Table 1) containing T7 promoters at the 5′ ends were used to amplify the HK, c-Myc, CREB and GFP coding regions (564, 499, 466 and 700 bp, respectively). Recombinant plasmids containing HK, c-Myc, CREB or GFP sequences were used as templates for PCR amplification. dsRNA transfections were performed using the FuGENE transfection reagent as described above, and cells were collected 24–48 h after RNAi; dsGFP served as a negative control.

### Chromatin immunoprecipitation (ChIP) assays

ChIP assays were performed as previously described [1]. Briefly, pupal brains were homogenized in 1 ml of nuclear extraction buffer (0.5% Triton X-100, 10 mM Tris-HCl (pH 7.5), 3 mM CaCl_2_, 0.25 M sucrose, 1 mM PMSF, and 1 mM DTT) and 37% formaldehyde was added to a 1% final concentration. The tubes were then rotated for 15 min at room temperature, sonicated, and the chromatin concentrations were quantified and equalized. Anti-POU, or -CREB or -c-Myc antibodies were used for immunoprecipitations, pre-immune serum served as a no antibody control, and an irrelevant antibody (anti-Har-actin) served as a mock control. The beads were washed five times following overnight rotation at 4°C, mixed with 100 μl elution buffer and then rotated at 65°C overnight to reverse the crosslinking. Finally, the DNA was purified and subjected to PCR analysis.

### ROS measurements

Intracellular ROS levels were determined by image analysis using the molecular probe 2′, 7′-dichlorodihydrofluorescein diacetate (DCFH-DA, Invitrogen). HzAm1 cells were washed and treated with 2-deoxy-D-glucose (DOG) for 48 h. The media was then removed and replaced with 2 μM DCFH-DA for 30 min. The cells were then washed twice in phosphate-buffered saline and lysed using NET buffer (50 mM Tris-HCl (pH 7.4), 5 mM EDTA, 1% Triton X-100, and 150 mM NaCl) [8]. Fluorescence values were recorded using a 96-well plate reader with 485 nm excitation and 535 nm emission wavelengths, and the values were then normalized to protein contents.

Day 1 nondiapause pupae were injected with DOG, and the brains were dissected in PBS 48 h after injection. The brains were incubated for 1 h in the dark in PBS containing 10 μM DCFH-DA, washed twice with PBS and then imaged using an OLYMPUS IX71 microscope.

## SUPPLEMENTAL DATA TABLE AND FIGURE


