# Supplementary material for: Hexokinase is a key regulator of energy metabolism and ROS activity in insect lifespan extension
Source: Aging (Albany NY). 2016 Feb 7;8(2):245–58. doi: 10.18632/aging.100885 (PMC4789580; doi:10.18632/aging.100885)
Supplement: Supplementary file 1 [file aging-08-245-s001.pdf]

SUPPLEMENTAL DATA

Table 1. **Oligonucleotides used for plasmid constructed, RNAi and qPCR**

(1) Primers for stepwise deletion plasmid constructs	
Conmmon R	CTCGAG GCCGTGTGCCACGCAGAATAC
HKPF1	GCTAGC GTATCACAAAGTCGATGCGATG
HKPF2	GCTAGC GAGCCCGTATAAAGGAAAGTTAG
HKPF3	GCTAGC GCATGCTGACGTACCTGAATG
HKPF4	GCTAGC GTGCATTTGATACAGTTCA
HKPF5	GCTAGC GTAGCTACGCTGAGCACGTGC
HKPF6	GCTAGC CGCTGACACACCCGTGGC
HKPF7	GCTAGC GGCGACTCTTTATTCGTAGGC
HKPF8	GCTAGC CAATATTACATGAAGGGCGTC
HKPF9	GCTAGC CATTGCGAGTGACGTCACG
(The Nhe I and Xho I site underlined in primers)	
(2) Primers for RNAi	
dsHK-F1	GGATCCTAATACGACTCACTATAGGCTCATCGTTGGCACTGGAAGC
dsHK-F2	CTCATCGTTGGCACTGGAAGC
dsHK-R1	GGATCCTAATACGACTCACTATAGGGTAGACGGAGCCATCGATGC
dsHK-R2	GTAGACGGAGCCATCGATGC
dsCREB-F1	GGATCCTAATACGACTCACTATAGGGCTCCTTCTGCACAGGTCCAATC
dsCREB-F2	GCTCCTTCTGCACAGGTCCAATC
dsCREB-R1	GGATCCTAATACGACTCACTATAGGCTTCTAGCAATGGACCAGGGAC
dsCREB-R2	CTTCTAGCAATGGACCAGGGAC
dsc-Myc-F1	GGATCCTAATACGACTCACTATAGGGAGGATTCCTTGAGGTTGACGC
dsc-Myc-F2	GAGGATTCCTTGAGGTTGACGC
dsc-Myc-R1	GGATCCTAATACGACTCACTATAGGCCTCCTCTGATTCACATGAGTC
dsc-Myc-R2	CCTCCTCTGATTCACATGAGTC
(The T7 promoters site underlined in primers)	
(3) Primers for qPCR	
HK-qF	GAGATGACGGAGCGCTGGAC
HK-qR	CCTCCAAACAGCAAGCCCATC

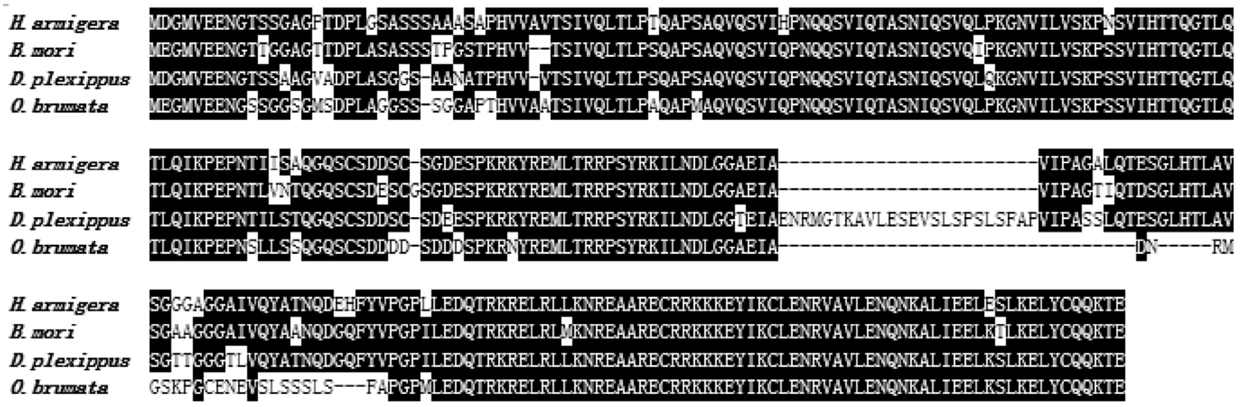

Figure S1. Homology comparison to other known CREB proteins. Black shading represents more than 50% sequence identity. *H. armigera, Helicoverpa armigera*; *B. mori, Bombyx mori; D. plexippus, Danaus plexippus*; *O.brumata, Operophtera brumata.*
